# The mediating roles of physical exercise and social-psychological stress in the relationship between socioeconomic status and self-rated health

**DOI:** 10.1371/journal.pone.0345542

**Published:** 2026-03-25

**Authors:** Xiaosheng Lei, Song He, Heng Jiang, Aijun Xu

**Affiliations:** 1 School of Management, Hubei University of Chinese Medicine, Wuhan, Hubei, P R China; 2 Hubei Research Center for the Development of Traditional Chinese Medicine, Wuhan, Hubei, P R China; 3 Huangshi Maternity and Children’s Health Hospital, Huangshi Key Laboratory of Birth Defects Prevention, Huangshi, Hubei, P R China; 4 Department of Public Health, School of Psychology and Public Health, La Trobe University, Melbourne, Victoria, Australia; 5 Melbourne School of Population and Global Health, University of Melbourne, Melbourne, Victoria, Australia; 6 School of Health Economics & Management, Nanjing University of Chinese Medicine, Nanjing, Jiangsu, P R China; University of Georgia, UNITED STATES OF AMERICA

## Abstract

**Objectives:**

Although the relationship between socioeconomic status (SES) and health is well documented, the contributions of physical exercise (PE) and social-psychological stress (SPS) to this association remain insufficiently understood. This study aimed to explore the mediating roles of PE and SPS in the relationship between SES and self-rated health (SRH).

**Methods:**

This study included 1,507 participants (aged ≥ 16 years) from the Health Survey conducted in Hubei Province during the post-pandemic period of 2021. Structural equation modeling (SEM) was used to examine the mediating and interaction effects, with PE and SPS specified as mediators in the association between SES and SRH.

**Results:**

The average SES and SRH scores were 3.07 ± 1.12 and 2.93 ± 0.93, respectively. Significant pairwise correlations (*p* < 0.05) were found among SES, PE, SPS and SRH. Low SES was associated with poorer SRH (β = 0.23), as were insufficient PE (β = 0.11) and higher SPS (β = –0.17). Both PE and SPS demonstrated significant mediating effects, accounting for 6.7% and 14.2% of the total impact of SES on SRH, respectively. The interaction between PE and SPS was statistically significant only among females (*p* < 0.05).

**Conclusions:**

This study confirms that physical exercise and social-psychological stress play partial mediating roles in the association between SES and SRH. The findings suggest that limited physical exercise and higher levels of social-psychological stress may adversely affect health, particularly with low SES. Promoting healthy lifestyles and supportive social environments is therefore crucial for improving population health.

## 1. Introduction

Although the relationship between socioeconomic status (SES) and health is well documented, the specific mechanism underlying this association remains insufficiently clarified. Based on the existing literature, four theoretical pathways have been proposed to explain how SES influences health: material structure, lifestyle, social psychological states, and the neighborhood environment [1–4]. However, how these pathways operate and through which factors they exert their influence still lack systematic empirical validation and require further investigation [4].

SES is recognized as a fundamental determinant of health in the social determinants of health (SDH) model [5–10]. SES encompasses multiple dimensions, including education, income, and occupation, all of which profoundly shape health status and well-being [11–15]. Higher income improves living conditions and access to health resources, whereas higher educational attainment enhances health literacy, and supports the adoption of preventive behaviors [16]. Empirical evidence consistently supports this association: Liang et al. (2023) and Xu et al. (2023) [17,18] found a positive correlation between SES and health among older adults, while An et al. (2025) reported that individuals with low SES faced a 1.5-fold greater risk of cardiovascular disease than those with high SES [9].

Among the potential mediators linking SES and health, physical exercise (PE) and social-psychological stress (SPS) have attracted increasing attention, as they represent key lifestyle and psychological pathways. Physical exercise, often described as a “healthy social vaccine,” can mitigate SES-related health disparities by improving physiological functioning and promoting social interaction [19–21]. Zhong et al. (2020) reported that individuals with higher SES tend to exercise more frequently, which contributes better health outcomes [19]. Bai et al. (2025) further verified that high SES combined with regular physical activity reduces the risk of cognitive decline [22].

Social-psychological stress serves as a “psychological bridge” in the SES-health relationship. Individuals with low SES are more likely to face resource constraints, increasing exposure to stressors that can result in both physical and mental health problems. According to the stress process model, social structural factors such as SES generate stressors, triggering anxiety, depression, and other stress responses that ultimately impair health [23]. During the COVID-19 pandemic, perceived stress was found to mediate the association between SES and adolescent mental health, and low-SES teenagers may experience higher levels of stress-related mental health issues [24]. Additionally, low SES has also been associated with an increased risk of depressive symptoms and insufficient physical activity [25,26].

Despite some studies on the SES-health relationship and the independent roles of PE and SPS, their combined contributions as mediators have not been adequately examined, and limited studies have explored gender differences in this multipath mechanism [4]. To address this gap, this study constructed a comprehensive SES index incorporating education, income and occupation and used data from the post-pandemic period to explore the roles of PE and SPS in the association between SES and self-rated health (SRH). Structural equation modeling (SEM) was employed to analyze both mediating and interaction effects of PE and SPS, with the aim of providing empirical evidence to inform targeted health promotion policies.

## 2. Materials and methods

### 2.1. Study framework and research hypotheses

**Fig 1:**
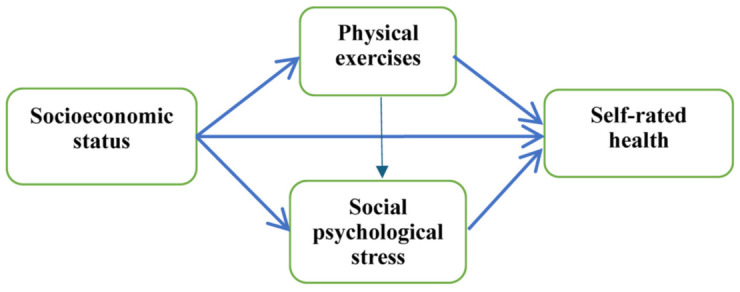
Mediation model between SES and SRH.

The theoretical mediating factors underlying the association between SES and health include material conditions, lifestyle, psychosocial factors, and the neighborhood environment. Among these, lifestyle and psychosocial factors are widely recognized as important mediators within the social determinants of health (SDH) framework. This study aims to investigate the mediating roles of PE and SPS in the relationship between SES and SRH. We hypothesized that both PE and SPS mediate the association between SES and SRH. The study framework is presented in Fig 1.

### 2.2. Study design and sample

A multistage cluster sampling method was employed in a health survey conducted across six cities in Hubei Province from May to August 2021. In each city, at least one urban community and one rural village were selected, and no fewer than 100 questionnaires were randomly distributed in each community. A total of 1,560 questionnaires were distributed, of which 1,507 valid responses were obtained. The inclusion criteria were as follows: (1) aged >16 years, and (2) provision of written informed consent and voluntary participation. The exclusion criteria were: (1) inability to complete the questionnaire independently due to severe illness and (2) unwillingness to participate. The study was approved by the Ethics Committee of Hubei University of Chinese Medicine (Approval number: 2024005).

The questionnaire was designed based on the social determinants of health (SDH) model, which posits that individual health is influenced by multiple factors, including personal characteristics, socioeconomic factors, health behaviors, and social-psychological stress. The questionnaire collected information on personal characteristics (gender, age, place of residence, and marital status), socioeconomic factors (annual income, education level, and occupation), health behaviors (frequency of physical exercise), social-psychological factors (measured using modified Chinese Perceived Stress Scale), and SRH.

### 2.3. Measures

#### 2.3.1. Socioeconomic status.

There are various approaches to measuring SES. Some studies utilize income and education as indicators, whereas others employ subjective SES measures [27]. However, most studies adopt a composite measure that integrates education, occupation, and income [28]. Li developed a composite SES scale incorporating these three indicators to measure social stratification in China [29]. The present study measured SES by integrating education, occupation, and income based on Li’s classification criteria.

In this study, SES was represented by three variables: personal income, education level, and occupation. Personal annual income was categorized into five groups, assigned scores from 1–5 points: “<30,000”, “30,000–50,000”, “50,001–100,000”, “100,001–200,000”, and “>200,000” Chinese yuan. Education level was classified into four groups: “primary school and below”, “junior high school”, “high school”, and “college and above”, with scores ranging from 1–4 points. In accordance with Li’s social class classification criteria [29], occupations were assigned scores from 1–4 points, representing low to high social class. The scores of all three indicators were summed to obtain a total SES score. The score ranged from 3 to 13 points. Based on this total score, SES was further categorized into 5 levels from low to high (Table 1).

**Table 1:** Descriptive statistics of the primary study variables.

Variables	Mean	Standard deviation	Range	Variable Definition
Socioeconomic status (SES)	3.07	1.12	1–5	5 groups from low to high
Frequency of physical exercise (PE)	2.17	2.21	0–7	Number of exercises per week, 0 = “no exercise”, 7 =” 7 or more times of exercises”
Social psychological stress (SPS)	16.16	3.84	8–32	Total score of CPSS scale
Self-rated health (SRH)	2.93	0.93	1–5	Score of 1–5 from low to high

#### 2.3.2. Self-rated health.

SRH was used as a health indicator. Although SRH is a subjective assessment, it is convenient to use and easily accepted by respondents. Moreover, extensive evidence confirms that SRH effectively predicts mortality and functional limitations, thereby serving as a valid proxy for objective health status [30,31]. Consequently, SRH is widely utilized in studies investigating the relationship between SES and health. Respondents rated their health on a five-point scale in the questionnaire: “very good”, “good”, “average”, “not good”, and “poor”, assigned values from 1 to 5 respectively. Higher scores indicated better SRH.

#### 2.3.3. Physical exercises.

Physical exercise was assessed as a key lifestyle behavior. The frequency of PE was defined as the number of physical exercise sessions per week, with a minimum of 30 minutes per session. A score of 0 indicated no physical exercise, scores from 1–6 represented the corresponding number of sessions per week, and a score of 7 indicated ≥7 sessions per week.

#### 2.3.4. Social-psychological stress.

Social-psychological stress was assessed by a modified Chinese Perceived Stress Scale (CPSS) revised by Yang [32], which is widely used in the Chinese population. The modified CPSS comprises eight items designed to assess stress levels, mental tension, and feelings of loss of control. Cronbach’s alpha coefficient in this study was 0.712, and the item-total correlation coefficient for all the items exceeded 0.5 (P < 0.001), indicating good reliability and content validity. Each item was rated on a four-point Likert scale, “never, rarely, sometimes, and usually”, with values ranging from 1 and 4. The total SPS score ranged from 8 to 32, with higher scores indicating greater levels of stress.

### 2.4. Statistical analysis

SPSS 19.0 software was used for data cleaning, descriptive statistics, and correlation analysis. AMOS 26.0 software was used to estimate the structural equation model (SEM). SEM is a statistical technique for testing complex theoretical models involving multiple variables. It was used to examine the mediating and interaction effects of PE and SPS on the association between SES and SRH. Based on these results, we can understand the relationships between variables and make informed decisions for health promotion. Statistical significance was denoted if *p* < 0.05.

## 3. Results

### 3.1. Sociodemographic characteristics

Table 2 describes the participants’ sociodemographic characteristics. The study included 1,507 participants, with 795 (52.8%) males and 712 (47.2%) females. Moreover, 53.4% of the participants resided in urban areas. A total of 1,129 participants (74.9%) were married, whereas 378 (25.1%) were not married, which included unmarried, widowed, and divorced participants. In terms of age distribution, 856 participants were under 45 years old (56.8%), 477 were aged 45–60 years (31.7%), and 174 were over 60 years old (11.5%).

**Table 2:** Respondents’ demographic characteristics.

Variables	Category	Number of samples	Percentage (%)
Gender	Male	795	52.8
	Female	712	47.2
Place of residence	Urban	804	53.4
	Rural	703	46.6
Marital status	Married	1129	74.9
	Unmarried	378	25.1
Age	<45	856	56.8
	45–60	477	31.7
	>60	174	11.5

### 3.2. Correlation analysis

Pearson correlation analysis was used to examine the relationships among SES, SPS, PE, and SRH. The results indicated significant positive correlations among SES, PE, and SRH (p < 0.05), as well as a significant negative association between SPS and the other three variables, SES, PE, and SRH (p < 0.05, Table 3).

**Table 3:** Correlation analysis between the study variables.

Variables	SES	SPS	PE	SRH
SES	1.000			
SPS	−0.163^**^	1.000		
PE	0.114^**^	−0.052^*^	1.000	
SRH	0.306^**^	−0.302^**^	0.207^**^	1.000

Note: ** denotes *p* < 0.01, * denotes *p* < 0.05. Abbreviations: SES, Socioeconomic status; PE, physical exercise; SRH, Self-rated health; SPS, Social-psychological stress.

### 3.3. Mediation analysis

The SEM was applied to investigate the role of PE and SPS in the relationship between SES and SRH. To control for potential confounding effects, occupation (farmer vs. non-farmer) was included as a control variable. The parameter estimates are presented in Table 4. The chi-square value of the model was 5.781, whereas the chi-square to the degree of freedom ratio (CMIN/DF), root mean square error of approximation (RMSEA), comparative fit index (CFI), normed fit index (NFI), and incremental fit index (IFI) were utilized to assess the model’s fit. The values of CMIN/DF = 2.891, and RMSEA = 0.036. All the CFI, NFI, and IFI values were > 0.90, suggesting a good fit of the model.

**Table 4:** Fitting indexes of the mediating model.

Fitting index	CMIN/DF	RMSEA	CFI	NFI	IFI	P
Reference value	<3	<0.08	>0.90	>0.90	>0.90	>0.05
Model value	2.891	0.036	0.994	0.991	0.994	0.056
Model fit	Yes	Yes	Yes	Yes	Yes	Yes

Note: Abbreviations: CFI, comparative fit index; CNIM/DF, chi-square to the degree of freedom ratio; IFI, incremental fit index; NFI, normed fit index; RMSEA, root mean square error of approximation.

#### 3.3.1. Effects of SES on SRH.

**Fig 2:**
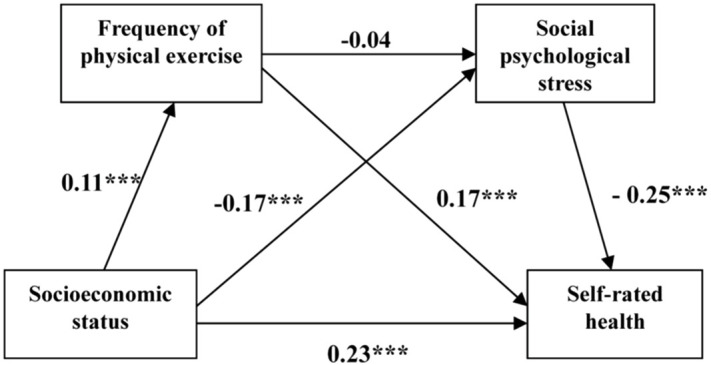
PE and SPS act as mediators in the association between SES and SRH.

Fig 2 shows that a one-unit increase in SES was associated with a 0.23-unit increase in SRH, indicating a positive correlation between SES and SRH. Individuals with higher SES trend to report better health, suggesting that SES plays an important role in explaining variations in health status.

#### 3.3.2. Mediating role of physical exercise.

Table 5 shows that higher SES was associated with an increased frequency of PE, and PE had a positive effect on SRH (*P* < 0.001). For every one-unit increase in SES, the frequency of PE increased by 0.11 units. Each unit increase in the frequency of PE was associated with 0.17-units increase in SRH. These results indicated that higher levels of PE contribute to better SRH. This may be because of that PE not only improves the body’s function and adaptability and reduces cardiovascular disease risk, but also alleviates stress, anxiety and depression, thereby promoting better mental health [33,34]. Therefore, the mediating role of PE in the association between SES and SRH was supported.

**Table 5:** Estimates of individual paths in the mediation model.

Independent variable	Dependent variable	Estimate	Standardized estimate	S.E.	C.R.	P
SES	PE	0.221	0.112	0.051	4.298	***
SES	SPS	−0.578	−0.168	0.089	−6.254	***
PE	SRH	0.073	0.173	0.010	7.271	***
SPS	SRH	−0.060	−0.248	0.006	−10.35	***
SES	SRH	0.194	0.234	0.022	8.789	***
PE	SPS	−0.062	−0.036	0.045	−1.371	0.17

*** denotes p < 0.001. Abbreviations: SES, socioeconomic status; PE, physical exercise; SRH, self-rated health; SPS, social-psychological stress.

#### 3.3.3. Mediating role of social-psychological stress.

The path coefficient revealed a negative correlation between SES and SPS as well as between SPS and SRH (*p* < 0.001). Specifically, for every one-unit increase in SES, SPS decreased by 0.17 units. Additionally, each unit increase in SPS was associated with a 0.25-unit decrease in SRH. This indicated that higher stress levels are linked to poorer health outcomes. This finding suggests that long-term SPS may lead to anxiety, depression, weakened immune system and an increased risk of disease, ultimately impairing quality of life and physical health [35]. Our results confirm that SPS acts as a mediator between SES and SRH, whereby SES influence health outcomes by reducing SPS.

The total effect of SES on SRH in the mediating model was 0.296, comprising direct and indirect effects of 0.234 and 0.062, respectively. PE positively affected SRH through SES, with an effect size of 0.020 (95% CI (0.005,0.035), *p* < 0.05), accounting for 6.7% of the total effect. SPS exerted a negatively mediating effect through SES on SRH, with an effect size of 0.042 (95% CI (0.015,0.065), *p* < 0.05), accounting for 14.2% of the total effect. Overall, the combined mediating effects of PE and SPS accounted for 20.9% of the total effect of SES on SRH.

#### 3.3.4. Interaction effect between social psychological stress and physical exercise.

**Fig 3:**
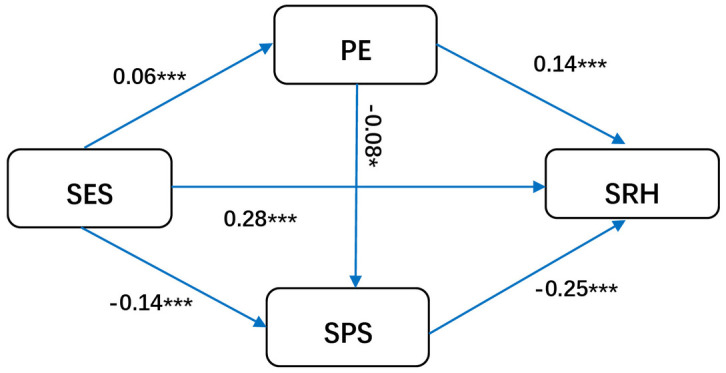
Mediation model for females (Abbreviations: SES, socioeconomic status; PE, physical exercise; SRH, self-rated health; SPS, social-psychological stress.).

**Fig 4:**
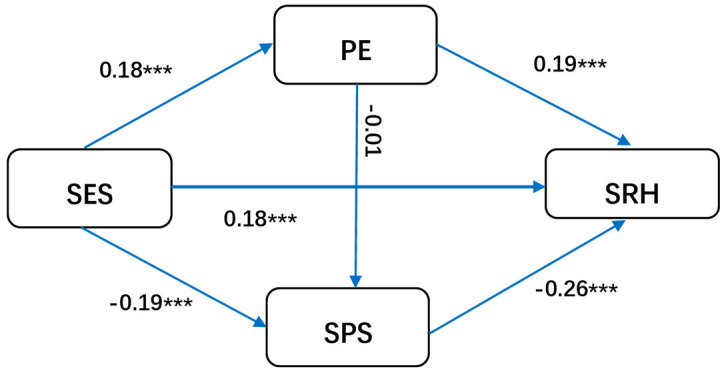
Mediation model for males (Abbreviations: SES, socioeconomic status; PE, physical exercise; SRH, self-rated health; SPS, social-psychological stress.).

The standardized estimate for the effect of PE on SPS was –0.016 (*p* > 0.05), indicating that the chain mediating effect between PE and SPS was not supported in the overall model. However, with respect to gender difference, when occupation was used as the control variable, an interaction was found between PE and SPS only in female group, with a standardized estimate of –0.08 (P < 0.05) (Fig 3). The positive effect of PE on SRH (*β =* 0.19, *P <* 0.001) was greater among males than females (*β* = 0.14, *P* < 0.001), whereas the negative effect of SPS on SRH was similar in males (*β* = –0.26, *P* < 0.001) and females (*β* = –0.25, *P* < 0.001) (Fig 4). The difference in the path coefficients between males and females was not significant according to multigroup analysis.

## 4. Discussion

This study investigated the roles of PE and SPS in the association between SES and SRH. The results revealed that SES had a significant positive effect on SRH. Furthermore, both PE and SPS functioned as partial mediators in the pathway from SES to SRH, accounting for 6.7% and 14.2% of the total effect of SES on SRH, respectively. Notably, this survey was conducted during the post-pandemic period. Due to epidemic-related restrictions, individuals’ opportunities for physical exercise were reduced, and social-psychological stress increased, which may explain why the mediating contribution of SPS was greater than that of PE. Consistent with the above findings, a significant positive correlation was observed between SES and PE, and higher frequencies of physical exercise were associated with better SRH. Conversely, SES exhibited a negative association with SPS, and elevated SPS levels corresponded to poorer SRH. These results collectively highlight the dual mediating mechanisms through which SES influences SRH.

SES is a fundamental determinant of health disparities, and individuals with lower SES are more likely to report poorer SRH. The present study further verifies that PE serves as a critical mediator in this relationship. From the perspective of social ecological theory, health behaviors (e.g., physical exercise) are shaped by social environmental factors such as SES, which in turn affect health outcomes. This aligns with the findings of Zhong et al. (2020), who utilized data from the China General Social Survey (CGSS) and reported that for every one-level increase in SES, an individual’s health score increased by 0.21 units (P < 0.001), and this association was more pronounced among women, rural residents, and individuals with low educational attainment [19]. Our study demonstrated that a one-level increase in SES was linked to a 0.11-unit increase in PE frequency and a 0.23-unit increase in SRH. These findings suggest that individuals with higher SES tend to engage in more frequent physical exercise, which contributes to improved health. This result is consistent with both daily life experience and previous research by Huang and Xia [36,37].

The current study also confirms that SPS mediates the SES-SRH relationship: higher SES was associated with lower SPS levels, which in turn predicted better SRH. This finding is supported by Zhang et al. (2025), who reported a significant negative correlation between childhood subjective SES and anxiety levels (r = –0.14, P < 0.01), with psychosocial stress acting as a key mediator [26]. Psychosocial stress in modern society stems from multiple sources, including work pressure, interpersonal relationships, financial hardships, and daily life challenges. Chronic exposure to such stress can severely impair both physical and mental health well-being, contributing to conditions such as anxiety, depression, and cardiovascular diseases [38–40]. SPS is negatively associated with health, especially in those with lower SES backgrounds [8,10,41]. Zhang further confirmed that social stress was negatively correlated with SES, with SPS being more common in people with lower SES [42]. Du et al. (2020) reported that cumulative negative life events mediate the association between SES and depression in older adults [43]. Effective stress management strategies such as physical exercise, relaxation techniques, and social support can mitigate the adverse health effects of SPS. For individuals with lower SES, adopting these strategies may help alleviate the health risks associated with chronic stress. Individuals with higher SES often possess stronger social connections and greater access to social capital [31,44], which enables them to expand social networks, obtain more social support, reduce negative emotional impacts, and ultimately improve health outcomes.

Regarding the interaction between PE and SPS, a significant effect was observed only in the female subgroup; no significant interaction was detected in the overall population or the male subgroup. This gender difference may be attributed to men’s greater physical activity in work and daily life, which could inherently buffer psychological stress and thereby weaken the role of PE in SPS. Therefore, the effect of PE on SPS was not significant among men. Additionally, the limited sample size may have constrained the statistical power to detect such interactions.

Despite the novel insights provided by this study, several limitations should be acknowledged. Firstly, the study relied primarily on self-reported data, which may be subject to recall bias and social desirability bias. Secondly, the cross-sectional design prevents the establishment of causal relationships between the variables, and longitudinal studies can better elucidate the temporal sequence and mediation processes in this model. Finally, the survey was conducted across six cities in Hubei Province during post-pandemic period, limiting generalizability across regions and time periods. Future studies with larger and more diverse samples are warranted.

## 5. Conclusions

The present study confirms that both PE and SPS serve as partial mediators in the association between SES and SRH. People with low SES may have a high risk of poor SRH. However, the study indicated that regular physical exercise and reduced social-psychological stress could buffer against the risk of poor health, especially for low-SES individuals.

These findings provide useful insights for developing targeted health policies, especially for individuals with low SES. Local governments and health departments could establish low-cost or free community fitness programs (e.g., morning exercise in the park or neighborhood walking groups). Employers could implement “active break” initiatives or flexible exercise time policies to encourage regular physical activity during work hours—building on successful examples such as the “100-Day Walking” campaign launched at Hubei University of Chinese Medicine in 2024 and the “Fitness and Weight Loss” initiative at Guangzhou University of Chinese Medicine, both of which effectively improved staff health. In addition, community-based psychological counseling services, stress management workshops, and peer-support programs could help individuals manage stress more effectively. A province-wide health literacy campaign—delivered through social media platforms (e.g., WeChat), television, and community health workers—could further raise public awareness of the benefits of regular physical exercise and stress reduction [45,46], particularly among lower-SES groups. These policy options collectively have the potential to mitigate SES-related health disparities and enhance overall population health and well-being in the post-pandemic era.

## Supporting information

S1 Fig Fig 2. (PDF)

S2 Fig Fig 3. (PDF)

S3 Fig Fig 4. (PDF)
