# Supplementary material for: The mediating roles of physical exercise and social-psychological stress in the relationship between socioeconomic status and self-rated health
Source: PLoS One. 2026 Mar 25;21(3):e0345542. doi: 10.1371/journal.pone.0345542 (PMC13016283; doi:10.1371/journal.pone.0345542)
Supplement: S1 Fig — (PDF) [file pone.0345542.s001.pdf]

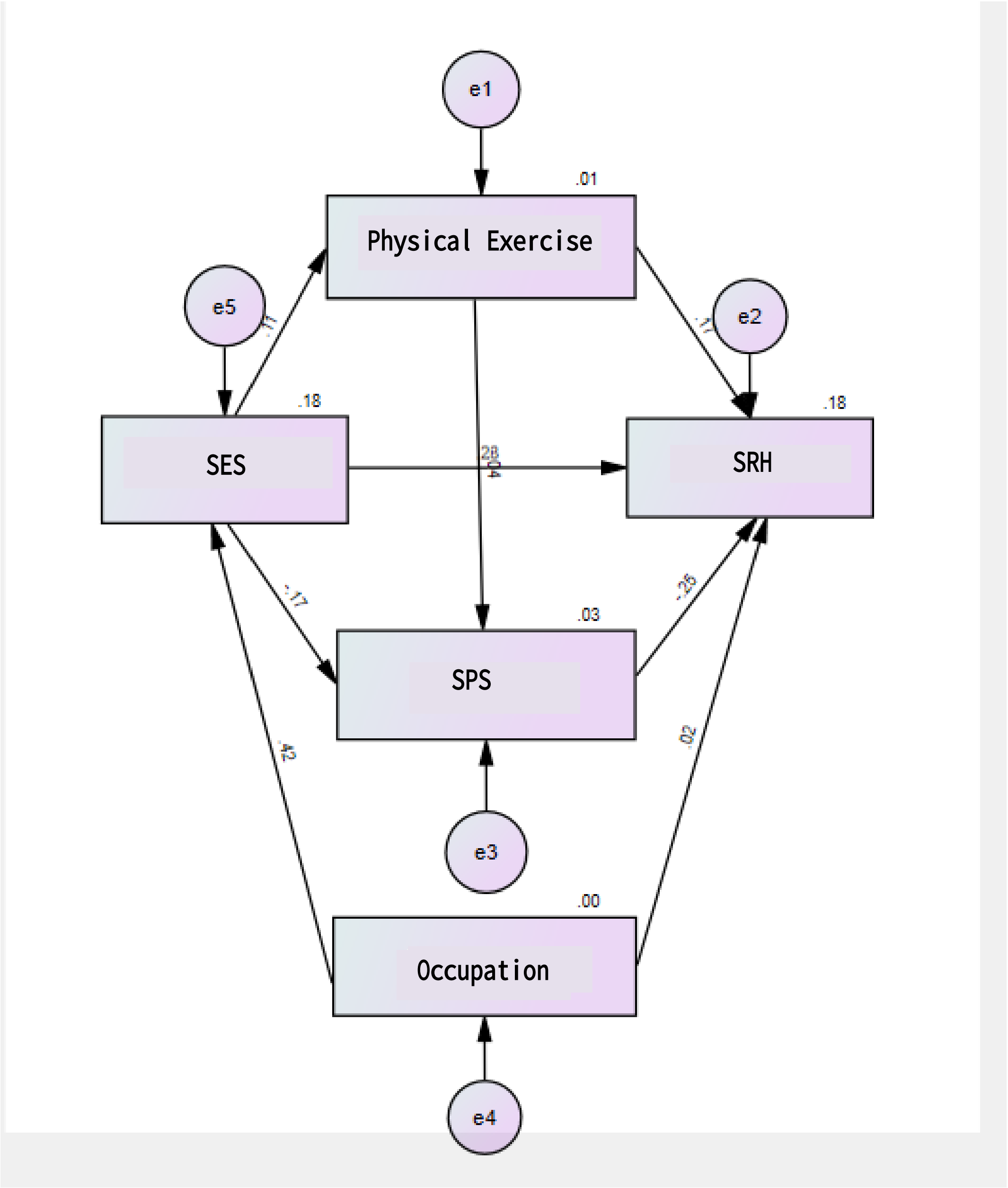

PE and SPS act as mediators in association between SES and SRH （take occupation as control variable）

Maximum Likelihood Estimates

Regression Weights: (Overall- Default model)

			Estimate	S.E.	C.R.	P	Label
SES	<---	Occupation	1.365	.077	17.736	***	par_7
PE	<---	SES	.221	.051	4.298	***	par_6
SPS	<---	PE	-.062	.045	-1.371	.170	par_4
SPS	<---	SES	-.578	.089	-6.488	***	par_8
SRH	<---	PE	.073	.010	7.271	***	par_1
SRH	<---	SES	.194	.022	8.789	***	par_2
SRH	<---	SPS	-.060	.006	-10.347	***	par_3
SRH	<---	Occupation	.049	.071	.693	.489	par_5

Standardized Regression Weights: (Overall - Default model)

			Estimate
SES	<---	Occupation	.424
PE	<---	SES	.112
SPS	<---	PE	-.036
SPS	<---	SES	-.168
SRH	<---	PE	.173
SRH	<---	SES	.234
SRH	<---	SPS	-.248
SRH	<---	Occupation	.018
