# Supplementary material for: The mediating roles of physical exercise and social-psychological stress in the relationship between socioeconomic status and self-rated health
Source: PLoS One. 2026 Mar 25;21(3):e0345542. doi: 10.1371/journal.pone.0345542 (PMC13016283; doi:10.1371/journal.pone.0345542)
Supplement: S2 Fig — (PDF) [file pone.0345542.s002.pdf]

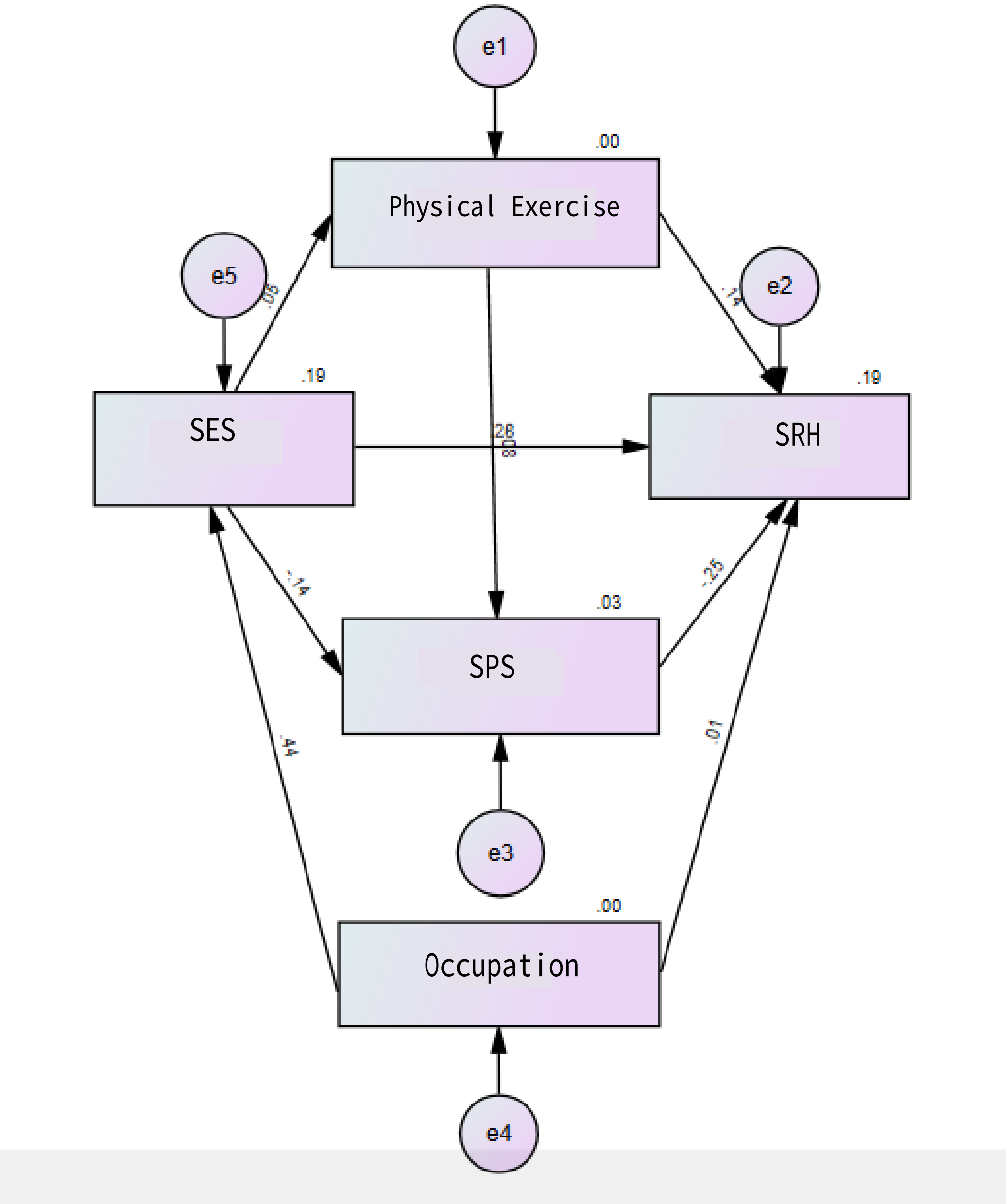

Fig 3. Mediation model for female (Original image in AMOS)

Take occupation as control variable

Estimates (Female - Default model)

Scalar Estimates (Female - Default model)

Maximum Likelihood Estimates

Regression Weights: (Female - Default model)

	Estimate	S.E.	C.R.	P	Label
SES <--- Occupation	1.415	.113	12.495	***	par_7
PE <--- SES	.106	.075	1.415	.057	par_6
SPS <--- PE	-.144	.066	-2.189	.029	par_4
SPS <--- SES	-.478	.127	-3.752	***	par_8
SRH <--- PE	.060	.015	4.116	***	par_1
SRH <--- SES	.226	.031	7.194	***	par_2
SRH <--- SPS	-.060	.009	-7.013	***	par_3
SRH <--- Occupation	.015	.101	.145	.884	par_5

Standardized Regression Weights: (Female - Default model)

	Estimate
SES <--- Occupation	.436
PE <--- SES	.055
SPS <--- PE	-.084
SPS <--- SES	-.144
SRH <--- PE	.144
SRH <--- SES	.281
SRH <--- SPS	-.248
SRH <--- Occupation	.006
