# Supplementary material for: The mediating roles of physical exercise and social-psychological stress in the relationship between socioeconomic status and self-rated health
Source: PLoS One. 2026 Mar 25;21(3):e0345542. doi: 10.1371/journal.pone.0345542 (PMC13016283; doi:10.1371/journal.pone.0345542)
Supplement: S3 Fig — (PDF) [file pone.0345542.s003.pdf]

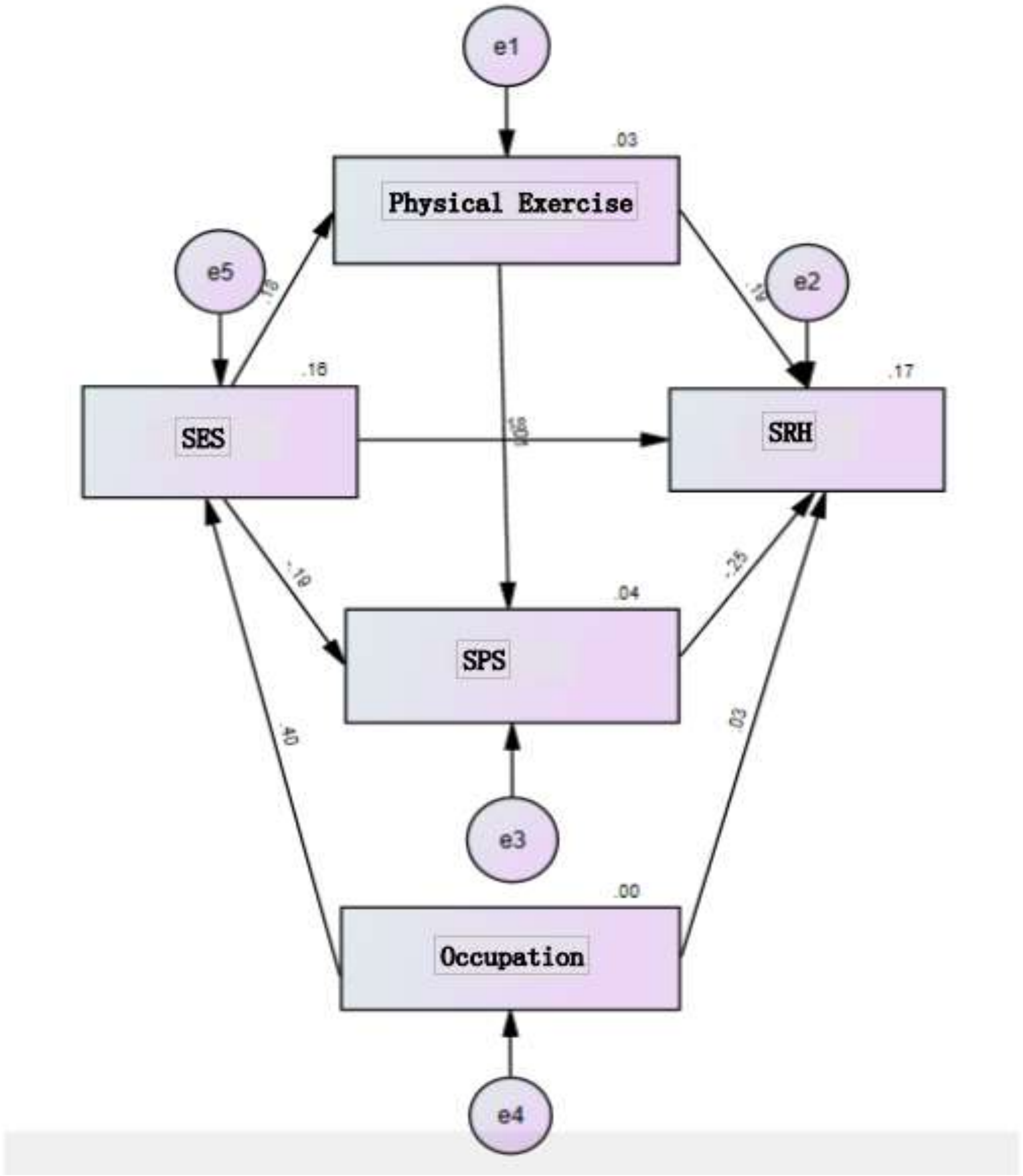

Fig 4. Mediation model for male (Original image in AMOS)

Take occupation as control variable.

Estimates (Male - Default model)

Scalar Estimates (Male - Default model)

Maximum Likelihood Estimates

Regression Weights: (Male - Default model)

			Estimate	S.E.	C.R.	P	Label
SES	<---	Occupation	1.252	.102	12.255	***	par_7
PE	<---	SES	.371	.074	5.015	***	par_6
SPS	<---	PE	-.024	.063	.382	.702	par_4
SPS	<---	SES	-.705	.132	-5.341	***	par_8
SRH	<---	PE	.080	.014	5.666	***	par_1
SRH	<---	SES	.158	.032	4.854	***	par_2
SRH	<---	SPS	-.061	.008	-7.622	***	par_3
SRH	<---	Occupation	.095	.098	.972	.331	par_5

Standardized Regression Weights: (Male - Default model)

			Estimate
SES	<---	Occupation	.405
PE	<---	SES	.178
SPS	<---	PE	-.014
SPS	<---	SES	-.192
SRH	<---	PE	.189
SRH	<---	SES	.179
SRH	<---	SPS	-.255
SRH	<---	Occupation	.035
